# Modelling Parasite Transmission in a Grazing System: The Importance of Host Behaviour and Immunity

**DOI:** 10.1371/journal.pone.0077996

**Published:** 2013-11-06

**Authors:** Naomi J. Fox, Glenn Marion, Ross S. Davidson, Piran C. L. White, Michael R. Hutchings

**Affiliations:** 1 Disease Systems Team, SRUC, Edinburgh, United Kingdom; 2 Environment Department, University of York, York, United Kingdom; 3 Biomathematics and Statistics Scotland, Edinburgh, United Kingdom; University of Ulm, Germany

## Abstract

Parasitic helminths present one of the most pervasive challenges to grazing herbivores. Many macro-parasite transmission models focus on host physiological defence strategies, omitting more complex interactions between hosts and their environments. This work represents the first model that integrates both the behavioural and physiological elements of gastro-intestinal nematode transmission dynamics in a managed grazing system. A spatially explicit, individual-based, stochastic model is developed, that incorporates both the hosts’ immunological responses to parasitism, and key grazing behaviours including faecal avoidance. The results demonstrate that grazing behaviour affects both the timing and intensity of parasite outbreaks, through generating spatial heterogeneity in parasite risk and nutritional resources, and changing the timing of exposure to the parasites’ free-living stages. The influence of grazing behaviour varies with the host-parasite combination, dependent on the development times of different parasite species and variations in host immune response. Our outputs include the counterintuitive finding that under certain conditions perceived parasite avoidance behaviours (faecal avoidance) can increase parasite risk, for certain host-parasite combinations. Through incorporating the two-way interaction between infection dynamics and grazing behaviour, the potential benefits of parasite-induced anorexia are also demonstrated. Hosts with phenotypic plasticity in grazing behaviour, that make grazing decisions dependent on current parasite burden, can reduce infection with minimal loss of intake over the grazing season. This paper explores how both host behaviours and immunity influence macro-parasite transmission in a spatially and temporally heterogeneous environment. The magnitude and timing of parasite outbreaks is influenced by host immunity and behaviour, and the interactions between them; the incorporation of both regulatory processes is required to fully understand transmission dynamics. Understanding of both physiological and behavioural defence strategies will aid the development of novel approaches for control.

## Introduction

Parasitic helminths present one of the most pervasive challenges to grazing herbivores [1]. The prevalence and intensity of parasite outbreaks is determined by a multitude of factors. These include the influence of host immunity on parasite establishment and fecundity, and the timing and frequency of contacts with parasites’ free-living infective stages. There is a propensity for macro-parasite transmission models to focus only on host immunological defence strategies, omitting more complex interactions between hosts and their environment [2]–[7]. This paper explores how host behaviours influence macro-parasite transmission in a spatially and temporally heterogeneous environment. The focus is on gastro-intestinal nematodes (GINs), transmitted via the faecal oral route, within a controlled grazing system.

Both nutritional resources and infective larvae are unevenly distributed in both space and time [8], [9]. Host grazing behaviours contribute to the generation of this heterogeneity, and are crucial in determining exposure to disease risk in a grazing system [10]. Through faecal avoidance it is believed that herbivores can limit contact with pathogens transmitted via the faecal-oral route, consequently lowering infection risk [11]–[15]. This selective grazing results in a heterogeneous resource distribution, with mosaics of tussocks (tall faecally contaminated patches), and gaps (short, faecally uncontaminated patches) [8]. As the contaminated tussocks harbour increased concentrations of both parasites and nutritional resources, the mosaic represents a parasitism versus nutrition trade-off [8], [16], [17]. The grazing behaviours of herbivorous hosts have been extensively studied [11]–[15], [18]–[20], allowing mathematical models to be meaningfully parameterised to encapsulate the grazing processes. In the model developed by Marion et al. [21] and Swain et al. [22] the behaviour of grazing herbivores in response to local environmental cues has been described using a spatially explicit model incorporating stochastic rules representing primary behavioural responses. This model, based on empirically observed rules of thumb, has been shown to simulate the nutrition versus parasitism trade off observed in grazing systems [21]–[23], and it has been demonstrated that the emergent properties of this model match empirical observations [23]. Within this framework, contacts with faecally-contaminated swards have previously been employed as a measure of potential infection events [23], [24] and parasite transmission was not explicitly incorporated.

In addition to the potential influence of grazing behavior, the host immune response plays a crucial role in transmission dynamics. Prolonged exposure to the infective stages of GINs leads to a decrease in the establishment, fecundity and survival of parasites in the host [25]–[30]. The incorporation of host immunity as a regulatory constraint of parasite populations within transmission models can explain key features of the dynamics [31], and its influence has been investigated previously, in the absence of host behaviour. Roberts & Grenfell [7] proposed a mechanistic model encapsulating the dynamics of directly transmitted GIN infections in managed ruminant populations. Their model captured key aspects of the parasite’s infrapopulation, suprapopulation, and the regulation of transmission through the host’s acquired immune response [7]. This deterministic model represented the average host (adult worm burden and immune response) and the infective stage larval population on an average sward. In an extension of this work, Marion et al. [32] developed a stochastic formulation of the model to account for dynamics at low population levels, and to incorporate variability and extinctions. GINs of livestock represent one of the best understood host-parasite systems and the life-cycles of GINs have been extensively studied. This allows models to be meaningfully parameterised to represent the lifecycles of GINs of grazing herbivores [4], [33]–[36].

Physiological and behavioural elements of transmission should not be considered in isolation, as there is a two-way interaction between host grazing behaviour and parasite burden. In grazing herbivores, parasitism can induce inappetence, reduction in grazing time and changes in grazing behaviour [37]–[40]. The extent of this parasite induced anorexia will vary with the degree of pathological changes and the parasites’ sites of predilection within the host [38]. This anorexia leads to intake reductions of between 30 and 60%, compared with uninfected animals [41]–[43]. Parasitised hosts also exhibit higher levels of faecal avoidance compared to uninfected grazers [8], [15]–[17], [40]. As resistance to infection is acquired, anorexia ceases and intake and faecal avoidance levels return to normality [8], [16], [42], [44]. It has been suggested that parasite-induced anorexia evolved to either facilitate host recovery or benefit the parasite, rather than merely being a maladaptive response of no benefit to either party [39], [45]. However, there is much debate over the function of anorexia [42], [46].

This paper develops a framework which integrates a stochastic version of the parasite transmission model of Roberts & Grenfell (1991) [7], with the grazing model of Marion et al. [21] to create a spatially-explicit, individual-based model that incorporates both the host’s immunological response to parasitism and key grazing behaviours. This integrated approach also incorporates the other pivotal elements of the transmission process: survival and development of the parasite both within the host and on pasture; spatial heterogeneity of both pathogens and resources; and the interactions between host grazing behaviour and parasitised state.

This framework is subsequently applied to explore how host behaviours influence macro-parasite transmission in a spatially and temporally heterogeneous environment, with the following objectives: 1) Determine how host parasite burden is influenced by spatial aggregation of both nutritional resources and infective larvae on pasture; 2) Determine the impact of host faecal avoidance behaviour on the timing and intensity of parasite outbreaks, for parasites with different on-pasture development times; 3) Determine the influence of faecal avoidance on parasite dynamics, for hosts with differing abilities to mount an immune response; and finally 4) Explore the interactions between host grazing behaviour and parasitised state, to elucidate potential benefits that anorexia can provide the host.

## Results

**Figure 1:**
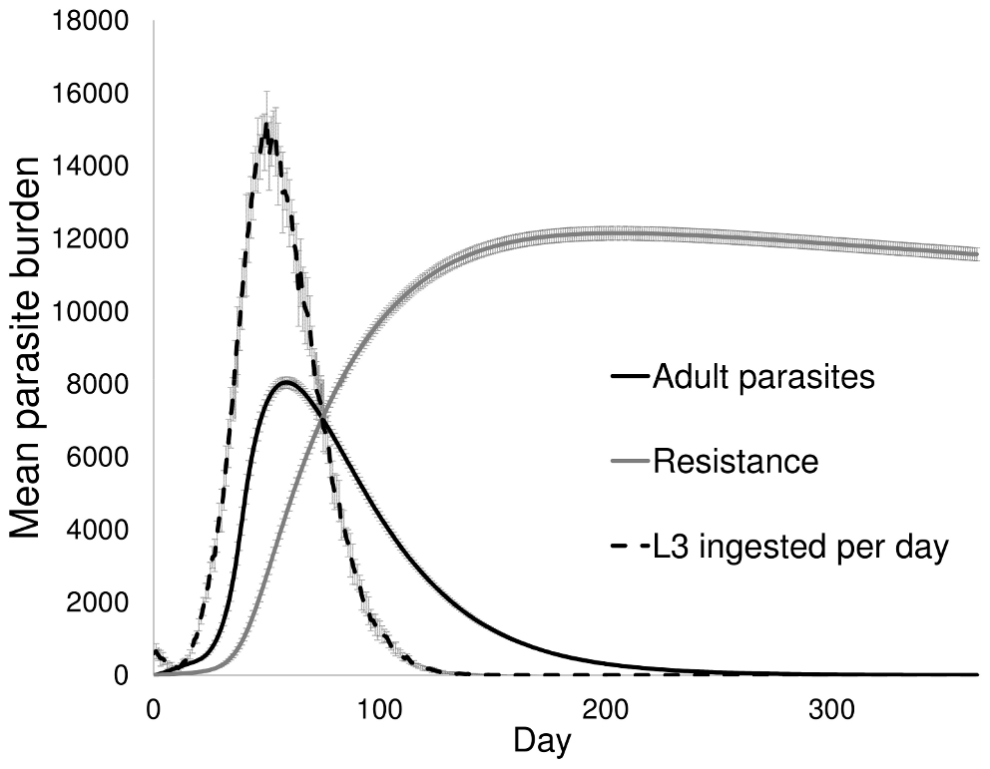
Parasite dynamics over one grazing season. Host parasite burden, L3 ingested per day and host resistance level (±SD) over one grazing season, using the standard parameter values detailed above (*μ_k_* = 3).

Using values outlined in the main parameterisation section, the model successfully reproduces the parasite dynamics empirically observed in livestock grazing systems [7], [47]–[50]. The introduction of susceptible hosts onto contaminated pasture accounts for the rapid increase in ingested larvae and adult parasites in the host, and the subsequent acquisition of immunity accounts for the consequent decline in parasite burden (Figure 1).

### Aggregation of Risk on Pasture

**Figure 2:**
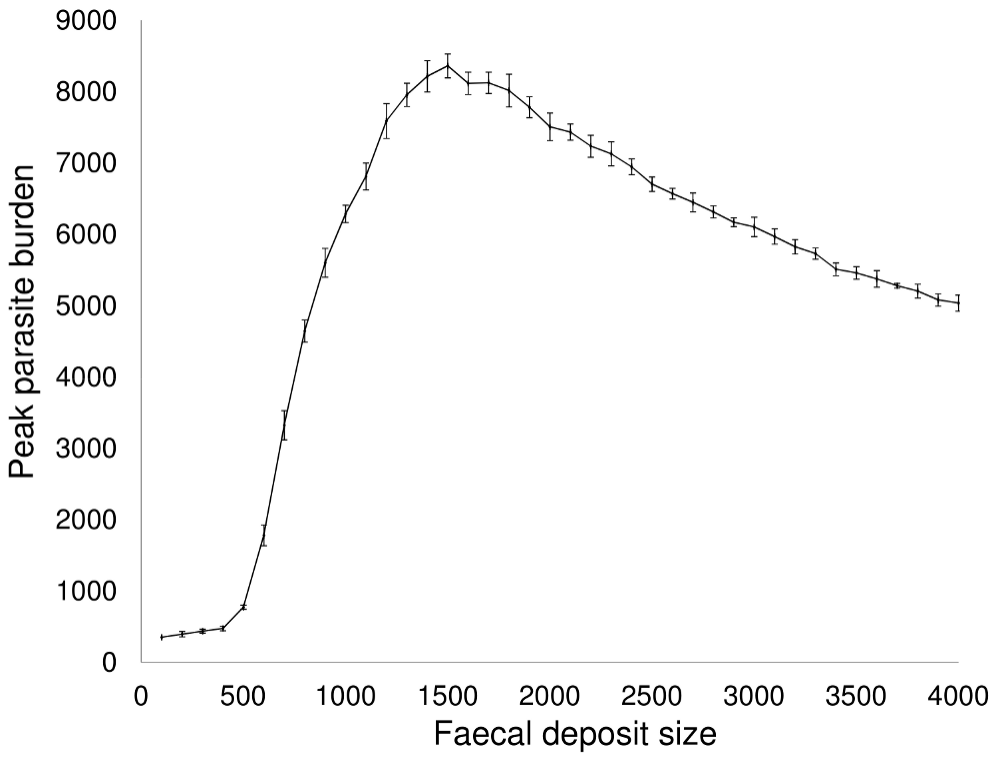
Influence of spatial clustering of both free-living larvae and faecal contamination on peak parasite burden. (*f_dep_* = 1.0, *s_0_* = 100,…., 4000, in increments of 100).

As the size of faecal deposits increases the level of clustering of larvae increases, leading to a rise in the severity of outbreaks for small to moderate clustering levels (Figure 2). However, at higher levels of clustering, the peak parasite burden steadily declines.

**Figure 3:**
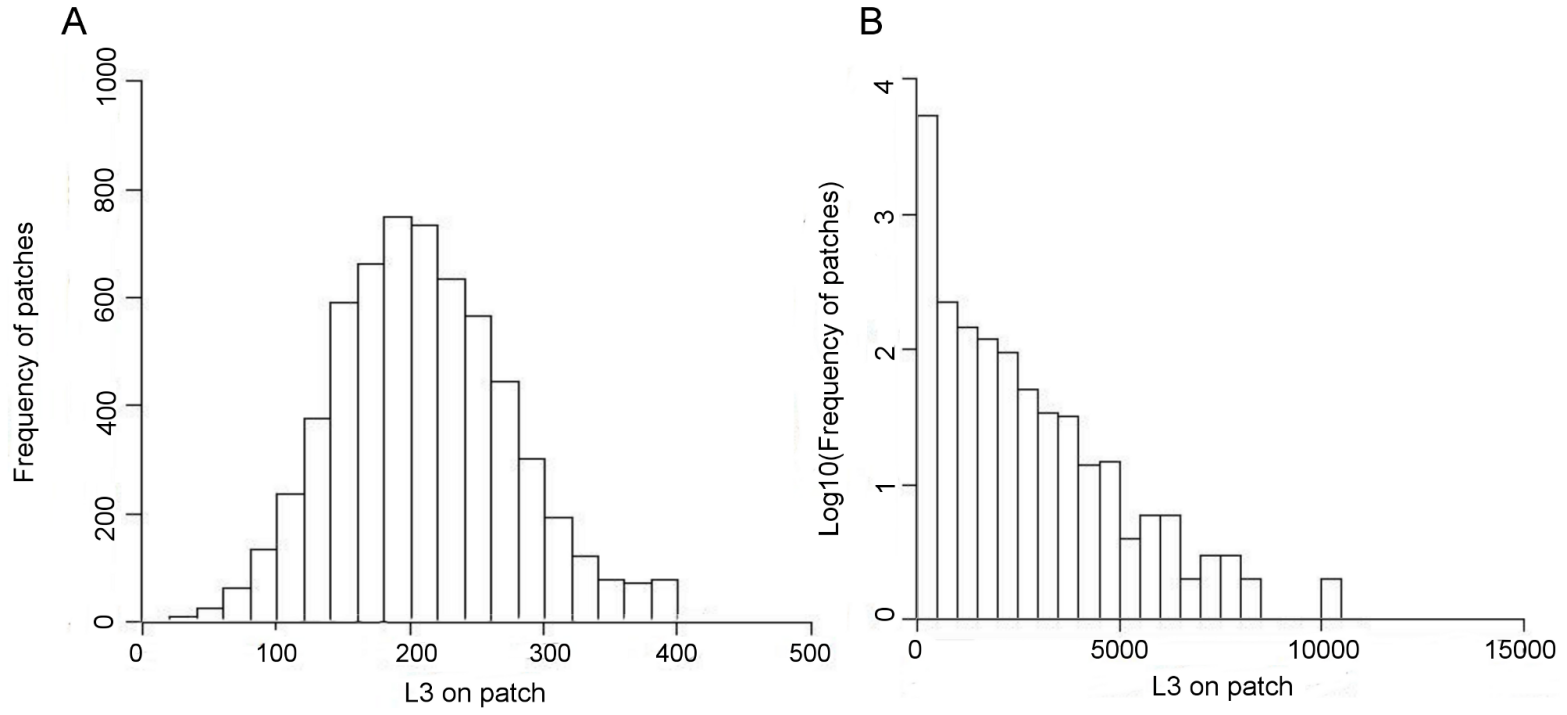
Distribution of L3 larvae on pasture at day 70. a) Low clumping scenario (*f_dep_* = 1.0, *s_0_* = 100; 1 faecal deposit per 100 bites) and b) realistic clumping scenario (*f_dep_* = 1.0, *s_0_* = 2000; 1 faecal deposits per 2000 bites). In figure b log10 of frequency of patches is used.

In runs with realistic levels of clustering (one faecal deposit per 2,000 bites [51]) spatial heterogeneity in infection risk is consistent with field observations [9] with larvae having a skewed distribution on pasture (Figure 3).

### Influence of Faecal Avoidance Across Parasites with Different Development Rates

**Figure 4:**
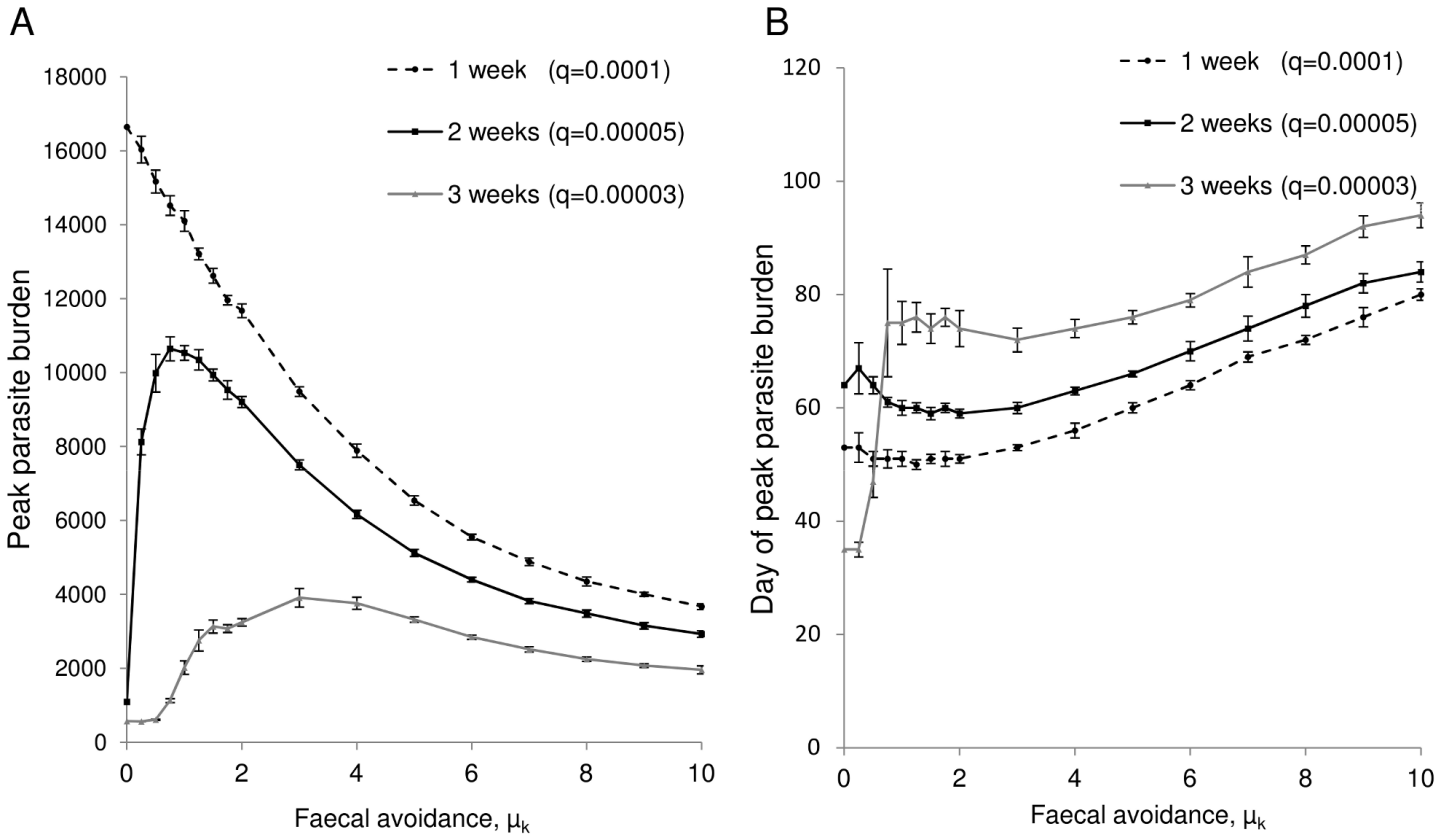
Impact of faecal avoidance and larvae on-pasture development time on parasite dynamics. a) Intensity and b) timing of peak parasite burdens over varying levels of faecal avoidance, for parasites with different development times on pasture. simulations were run with varying development rates (*q* = 0.00003 (development time of 3 weeks), *ε* = 0.00005 (development time of 2 weeks) and *q = *0.0001 (development time of 1 week), over differing faecal avoidance levels ranging from no avoidance (*μ_k_* = 0), to complete avoidance (*μ_k_* = 10).

Grazing behaviour also has a great influence on parasite transmission affecting both the timing and level of exposure to the parasite’s free living stages. For directly-transmitted pathogens that are infective immediately or develop quickly on pasture, faecal avoidance can decrease risk (figure 4). However, for parasites with delayed development on pasture, higher levels of faecal avoidance can lead to increased levels of parasitism (Figure 4a). For levels of faecal avoidance observed on pasture (*μ_k_* = 3 to 8) [24], [52]; this behaviour can lead to higher levels of risk from parasites which take over two weeks or longer to reach their infectious stage. In addition to influencing the magnitude of parasite burdens, faecal avoidance behaviour also affects the timing of outbreaks. As figure 4b shows, the higher the level of faecal avoidance the later the peak in burden.

### Influence of Faecal Avoidance Across Hosts with Different Rates of Resistance Acquisition

**Figure 5:**
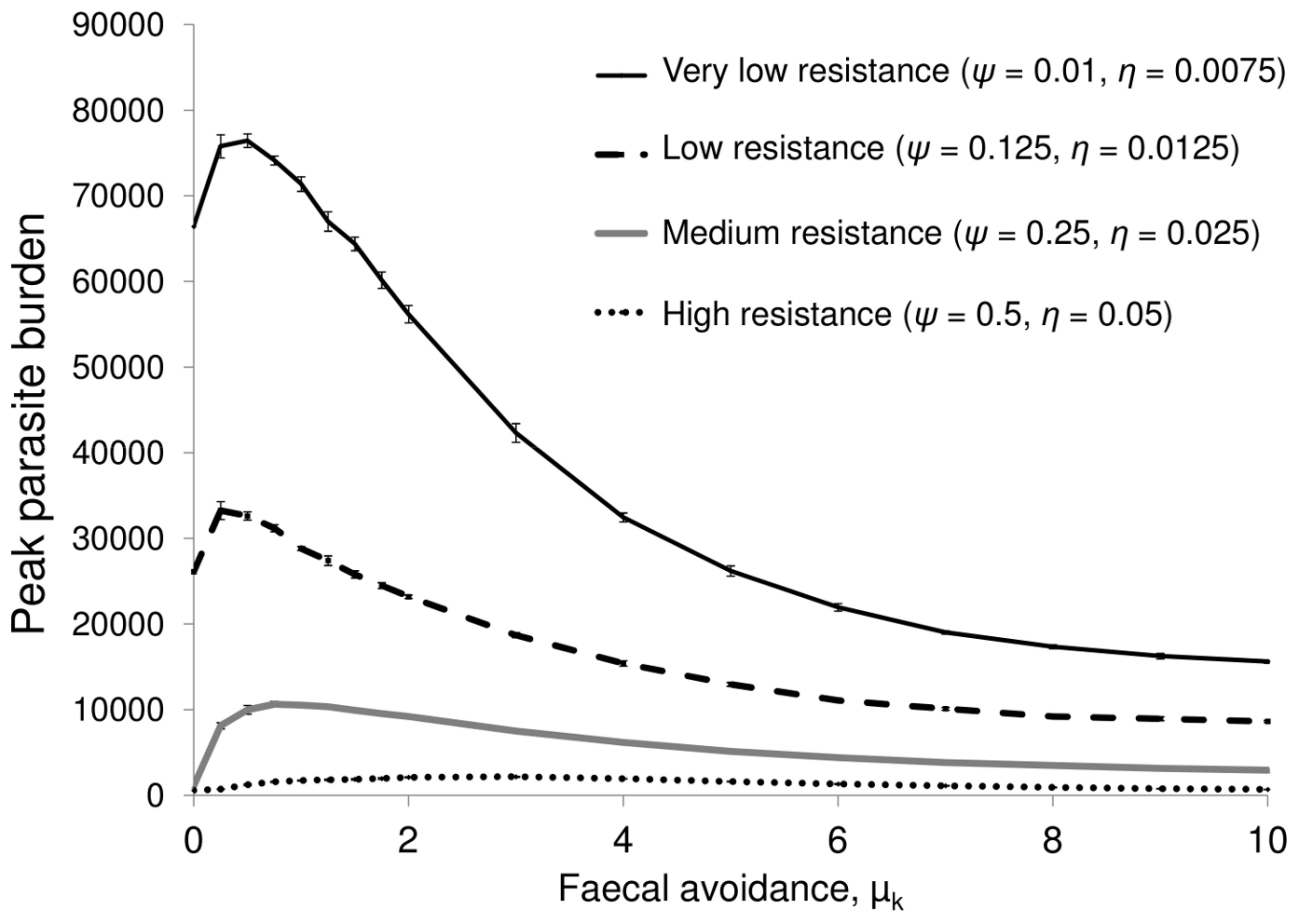
Peak parasite burden across varying faecal avoidance and host resistance acquisition levels. For a parasite that takes two weeks to develop on pasture, for cohorts of hosts with: very low resistance (*ψ* = 0.01, *η = *0.0075), low resistance (*ψ = *0.125, *η = *0.0125), medium resistance (*ψ = *0.25, *η = *0.025), high resistance (*ψ = *0.5, *η = *0.05). For each resistance level, simulations were run over differing faecal avoidance levels ranging from no avoidance (*μ_k_* = 0), to complete avoidance (*μ_k_* = 10).

The efficacy of faecal avoidance in minimising parasite risk varies with the host’s ability to mount an immune response. For hosts with a very limited ability to gain resistance, a range of faecal avoidance levels are advantageous (Figure 5).

Showing no faecal avoidance is a preferable strategy for parasite-host combinations where the host has an effective immune response and for pathogens with long on-pasture development times. In contrast, faecal avoidance is an effective defence strategy for parasite-host combinations where the host has limited ability to mount an immune response and for parasites with quick development times on pasture (figures 5 and 4a). However, increases in faecal avoidance can lead to decreases in daily herbage intake; this presents the hosts with a parasitism versus nutrition trade off.

### Parasite-host Interactions (Parasite Induced Anorexia)

To begin to explore the interactions between grazing behaviour and parasite dynamics, in the previous simulations grazing behaviour was conditionally independent of parasite burden (figures 1–5). In reality, individuals exhibit anorexia (increasing faecal avoidance and reduced daily intake) as parasite burdens rise, with grazing behaviour returning to normality once parasites are purged.

**Figure 6:**
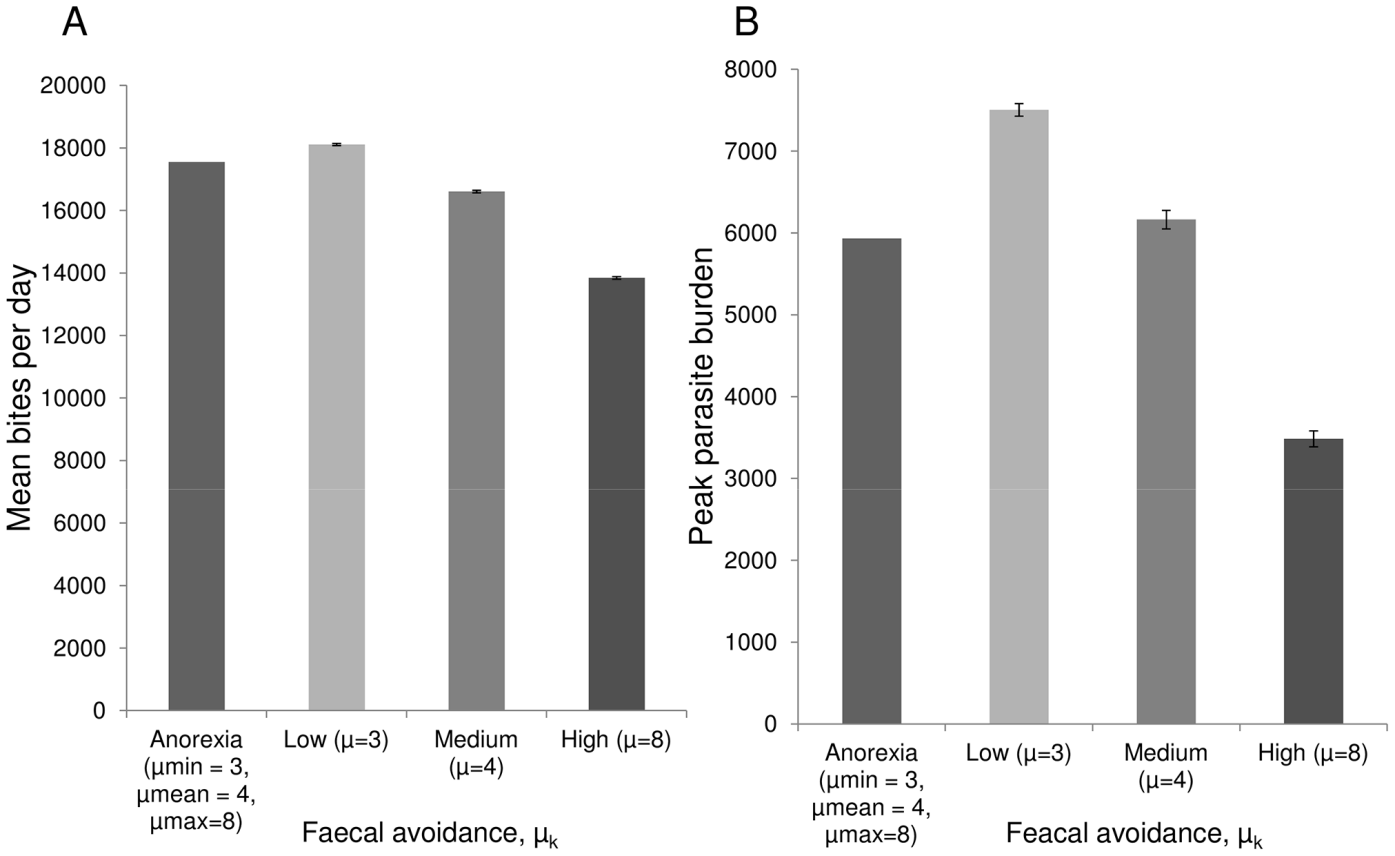
Influence of parasite-induced anorexia on herbage intake and peak parasite burden. Mean daily intake over one grazing season (a), and peak parasite burden (b), for different faecal avoidance strategies. Hosts with phenotypic variation in faecal avoidance leading to parasite-induced anorexia (with *μ_k_* = 3, *Λ* = 0.0006, such that min *μ_k_* = 3, max *μ_k_* = 8, and mean *μ_k_* = 4), and hosts with constant levels of faecal avoidance: low faecal avoidance (*μ_k_* = 3, *Λ* = 0), average faecal avoidance (*μ_k_* = 4, *Λ* = 0). ), and high faecal avoidance (*μ_k_* = 8, *Λ* = 0).

Figure 6 shows mean daily intake and peak parasite burden over one grazing season for hosts with phenotypic plasticity whose faecal avoidance is dependent on their parasite burden, and hosts with constant levels of faecal avoidance. Hosts which undergo an anorexic episode in response to parasite burden can benefit most from the nutrition versus parasitism trade-off (figure 6), minimising both parasite infection intensity and intake losses over the grazing season.

## Discussion

The infection dynamics shown in Figure 1 match the findings of Roberts and Grenfell [7], and the trends echo empirical data [47]–[50], with peak parasite burdens within realistic bounds [30], [47], [53]. The model also reproduces the grazing behaviours empirically observed at multiple scales in livestock grazing systems [40], [17], [54], [23] At the start of the grazing season, infection is initiated through ingestion of infective larvae that have over-wintered on pasture. Burdens initially stay at low levels as parasites in the host mature into fecund adults and the free-living stages develop into their infective state. Infections and pasture contamination then rise rapidly to a peak. This is followed by a precipitous decline as acquisition of resistance both reduces post exposure parasite establishment in individual hosts, and also reduces parasite fecundity thus regulating burdens at the supra-population level.

Transmission dynamics are influenced by processes which regulate infection (maintaining parasite population density within certain bounds), and those which control infection (perturbatory processes) [55]. For helminth-ruminant interactions host immune response is an important regulator of seasonal transmission dynamics within managed systems. Additionally, host grazing behaviour can control the timing and intensity of outbreaks.

### Aggregation of Risk on Pasture

Transmission dynamics are influenced by the spatial heterogeneity that is created and maintained in the system; the clumped release of host faeces and parasite progeny, and the host’s selective grazing, create uneven distributions of resources (grass), risk (infective larvae) and perceived risk (faecal contamination) on pasture.

For simulations parameterised with realistically-sized faecal deposits, spatial heterogeneity in infection risk is qualitatively consistent with field observations (figure 3b) [9]. High levels of aggregation increase the likelihood of high intensity parasite outbreaks (figure 2). At low clumping (a relatively even distribution of larvae on pasture), there are many exposure events, but at each event only a small number of larvae are ingested. This low-level trickle infection is enough to engender an immune response, but not to lead to high parasite burdens. As the level of clumping increases the skewness of the distribution of infective larvae on pasture increases, and the number of larvae ingested at each exposure event rises, allowing significant numbers of larvae to establish, resulting in high intensity outbreaks. It is worth noting that, although not incorporated in the model, aggregation also increases parasite mating probability within the host. For dioecious helminths there is a ‘breakpoint’ below which low mating frequency impedes transmission [31]. As levels of clustering increase beyond realistic values, the peak parasite burden steadily declines due to the presence of a decreasing number of more highly-contaminated patches. The severity of the faecal contamination cue in these patches, combined with the abundance of uncontaminated sward elsewhere, results in hosts only grazing these patches once the number of infective larvae in them has receded.

### Influence of Faecal Avoidance Across Parasites with Different Development Rates

In addition to influencing the spatial distribution of parasites, grazing behaviour also alters the timing of ingestion of the parasites’ free living stages. Hosts with no faecal avoidance encounter parasites when they are fresh on pasture, whilst increasing levels of faecal avoidance delay contact. For parasites that are immediately infective, or have quick development times on pasture, faecal avoidance decreases infection risk, as the host is less likely to graze contaminated patches when the population of infectious larvae is at its highest.

However, for parasites with delayed development on pasture (2 to 3 weeks), hosts without faecal avoidance have the lowest parasite burdens (figure 4a). This is because hosts without aversion to highly contaminated sward ingest a proportion of non-infective larvae soon after release onto pasture, decreasing the future potential population of infective larvae. This supports findings that parasite transmission can be reduced by co-grazing cattle with a second non-susceptible herbivore species, exploiting the parasite’s host specificity and enabling potentially infective larvae to be removed from the system [56].

Hosts with higher faecal avoidance levels take more bites from verdant patches once faeces has decayed, grass has grown tall and larvae have developed into their infective stage. This illustrates how faecal avoidance can increase parasite risk, and that faecal contamination level alone is not a reliable proxy for infection potential. This has previously been demonstrated by Van Der Wal et al. [57],who found that reindeer preferentially graze denser habitats where forage quality and quantity are greatest, but also where parasite infection risk is highest, and avoid drier sites with higher levels of dung deposition but smaller infective larvae populations [57].

The increased parasite risk incurred through faecal avoidance as demonstrated here could be amplified by environmental factors not currently included in the model. For example, contaminated patches that have been left ungrazed for an extended period would enable greater survival of L3 due to increased protection from heat and desiccation. A corollary to this has been demonstrated as a decrease in parasite intensity in cattle co-grazed with sows; the sows’ rooting behaviour breaks up the cattle faeces, reducing survival and availability of infective larvae [58].

### Influence of Faecal Avoidance Across Hosts with Different Rates of Resistance Acquisition

The extent to which grazing behaviour influences parasite transmission varies with the host’s ability to mount an immune response (figure 5). Development of immunity is affected by multiple factors including host age, nutritional and hormonal status, genotype and the influence of intercurrent diseases [27]. For hosts with limited ability to gain resistance, faecal avoidance can be advantageous (hosts with low levels of resistance acquisition, and faecal avoidance above µ_k_ = 2**_,_** have lower burdens than those with no avoidance, see figure 5). For hosts with impaired immunity and low faecal avoidance, low-level trickle infection from patches where some larvae have developed is enough for parasite establishment, but not for mounting an effective immune response, leading to high parasite burdens.

High levels of faecal avoidance can reduce parasite intensity for parasite-host combinations where the host has low levels of resistance acquisition (figure 5). However, high levels of faecal avoidance are potentially detrimental as the host’s ability to ingest enough nutrients would be greatly impaired in a set stocking scenario. Weight loss and inappetence have been observed in cows grazed on pasture with high levels of faecal contamination [59]. This highlights the trade-off between forage intake and parasite risk.

For hosts with a greater ability to mount an immune response, the low-level trickle infection received by hosts with no faecal avoidance is enough to engender an immune response, but not enough to lead to high levels of parasite establishment. If these hosts had high levels of faecal avoidance and delayed their encounters with infected patches until all larvae on that patch had matured, they would ingest large numbers of fully-developed larvae in one go, which could allow significant numbers of parasites to complete their lifecycle. This effect of faecal avoidance on parasite risk will vary across parasite species with different development times (see figure 4a).

In addition to changing the intensity of parasite outbreaks, grazing behaviour also affects the timing of peak parasite burdens (figure 4b). Faecal avoidance changes the timing of when hosts come into contact with parasites on pasture; this delay in L3 ingestions can delay the acquisition of immunity resulting in the parasite burden peaking later in the grazing season. This could have substantial consequences for production, as delaying the acquisition of immunity can lead to pathogenic parasitism shifting towards the time when livestock are older and normally productive [26]. As host susceptibility varies over the year with age and physiological status [44], [49], [60], changes in the timing of infection could further alter transmission dynamics. The timings of heavy infections (with regard to the age of the host) have also been shown to influence how the host is affected [60].

### Parasite-host Interactions (Parasite Induced Anorexia)

In the initial simulations (figures 1–5), host grazing behaviour was not explicitly dependent on parasite burden. In reality there is a two-way interaction between infection dynamics and grazing behaviour, with increased parasite intensity leading to reduced intake through increased faecal avoidance and a reduced bite rate. It has been suggested that anorexic behaviour can be of benefit to the host [42], [45], [61], however this benefit had not previously been demonstrated or quantified. Figure 6 shows the potential benefits that phenotypic plasticity in grazing behaviour can provide the host. Over the grazing season hosts that undergo an anorexic period can control their parasite burden with minimal loss of intake compared to hosts with fixed levels of avoidance.

Our findings qualitatively demonstrate the influence of anorexia on transmission dynamics. However anorexia is part of the generic acute phase response common to most infections [45], [61] and the potential costs and benefits are likely to vary across different pathogens and hosts. Specific parameterisation will be required to quantify the effects of anorexia in different systems. The inclusion of anorexia in the model does not allow for potential interactions between nutritional intake and the immune response. Associations between poor nutrition and infection levels have been demonstrated [44], [62]–[64]. Therefore, the benefits of anorexia shown here could be overestimated. However, parasitised hosts graze more selectively, selecting herbage with higher nutrient contents [44], so the short term decline in bulk herbage intake may not be mirrored by an equal decline in nutrient intake.

### Conclusions

In conclusion, grazing behaviour affects the timing and intensity of macro-parasite outbreaks, by generating spatial heterogeneity and changing the timing of exposure to the parasites free living stages. The influence of grazing behaviour varies with the host-parasite combination, with faecal avoidance behaviour being most beneficial when hosts have a limited ability to mount an immune response, and against parasites with fast on pasture development times. For macro-parasites with prolonged development times on pasture, faecal avoidance behaviour can increase risk. Further development of the model to incorporate co-infection with parasite species which exhibit varying development times could reveal an optimal grazing strategy. Our results also indicate that parasite-induced anorexia can be beneficial for the host through minimising both intake losses and parasite burdens over a grazing season.

Transmission models usually focus on the role of host immunity in regulating parasite dynamics. Our results illustrate that timing and magnitude of parasite outbreaks is driven by a combination of both grazing behaviour and host immunity, and the interactions between these regulatory processes. The structure of the model will facilitate the exploration of different control strategies; from chemotherapeutic applications and breeding for host resistance, to changes in grazing management. Manipulation of behavioural responses via grazing management could in many cases enhance existing intervention strategies. Furthermore, understanding the importance of both regulatory processes could aid the development of novel approaches for control. This integrated approach will also allow more informed predictions to be made about how outbreaks will be affected by future changes in the system.

## Methods

### Model Structure

Individual grazing is incorporated as in the model developed by Marion et al. [21] and further developed by Swain et al. [22] and Smith et al. [23],which incorporates the key elements of grazing behaviour and resource use in response to local environmental cues, and the outputs of which have been shown to match empirical observations [23]. The current study builds on this grazing model to incorporate pathogen population dynamics, both on pasture and within the host. A cohort of D animals (labelled *k* = 1…D) move around a lattice of N patches (labelled *i* = 1…N), making grazing decisions based on the sward height *h_i_* at that patch and the level of faecal contamination *f_i_*. The patch and animal state variables are outlined in table 1. All state variables within the model are assumed to be integers.

**Table 1:** State variables for patches, and animals.

Patch states	Notation
Co-ordinates of patch *i*	(*x_i_*, *y_i_*)
Sward height at patch *i*	*h_i_*
Faecal contamination at patch *i*	*f_i_*
Pre-infective larvae at patch *i*	*l_i_*
Infective L3 larvae at patch *i*	*L_i_*
**Animal states**	**Notation**
Location of animal *k*	*i_k_*
Immune response of animal *k*	*r_k_*
Immature parasites in animal *k*	*a_k_*
Mature parasites in animal *k*	*A_k_*
Parasite eggs in animal *k*	*e_k_*
Stomach contents of animal *k*	*s_k_*
Faecal deposit size	*s_0_*

Swain et al. [22] further developed the grazing model of Marion et al. [21] to explore the influence of search rate and search distance on host grazing. Following Swain et al. [22], the rate of movement from patch *i* to patch *j* is modelled as 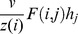, where *v* is the intrinsic movement rate and *h_j_* is the sward height at patch *j*, using the normalisation factor:
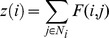



The search kernel *F(i,j)* follows the power-law 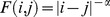 in which 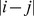 is the Euclidean distance between patch *i* and *j*. The normalisation prevents animals accumulating near the boundaries by avoiding lower movement rates at the boundary. If the search coefficient, *α*, is large, animals are restricted to nearest neighbour movement, while if *α* = 0 animals will search the whole lattice uniformly. In addition the total movement rates remain constant as *α* changes.

Sward growth is modelled logistically with the rate of increase at patch *i* given by:
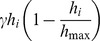
where *γ* is the intrinsic growth rate of the sward and *h_max_* is the maximum sward height attainable. The sward height of a given patch is reduced by *B* when an animal grazes at that location, while the stomach content *s_k_* of the corresponding animal is increased by one unit of size *B*. An individual takes a bite on its current patch at a rate:



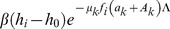
where *f_i_* represents the level of faecal contamination at patch *i*, *µ* is the level of faecal avoidance, *a_k_+A_k_* is the total number of parasites in host *k*, *Λ* is the anorexia coefficient, and h_o_ is the minimum grazable portion in each patch. Thus the bite rate is monotonically decreasing with the amount of faecal contamination and level of avoidance, and non-zero values of *g* allow for the avoidance to be amplified with increased parasite burden. The model also includes a daily intake requirement *R_k_* for each animal (as introduced by Smith et al. [23]). The intake of each animal accumulates until its requirement *R_k_* is reached, and is reset at the end of each day.

Grazing behaviour affects the timing of host contact with the parasites’ free living stages. To understand the interactions between grazing behaviours and parasite transmission, it is important to consider the multiple delays in the development of monoxenous nematodes. After release from the host the non-infective free living parasites (termed *l_i_* here) develop through multiple larval stages before reaching their infective third stage (L3) (termed *L_i_* here). After ingestion by an herbivorous host, they moult and develop onto fourth stage larvae (L4), before maturing into fecund adults (L5) [14]. The Roberts and Grenfell model [7] makes the implicit assumptions that larvae are instantaneously infective upon release onto pasture and parasites in the host are immediately fecund upon establishment. It is straightforward to relax these assumptions within the stochastic framework adopted here. Thus each patch (labelled *i* = 1…N) is assigned a number *l_i_* of pre-infective larvae as well as a number *L_i_* of infective L3 stage larvae. Similarly, within each host (labelled *k* = 1…D) separate variables *a_k_*, *A_k_* and e*_k_* are introduced for the number of immature parasites, mature parasites and eggs respectively. Incorporating these developmental delays [14], allows us to investigate the influence of grazing behaviour on parasite risk and the timings of outbreaks.

When an animal takes a bite of size *B*, the number of non-infective (*l_i_*) and infective larvae (*L_i_*) on its current patch, decrease by:
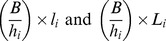



When an animal takes a bite of size *B*, the number of immature parasites in host *k, a_k_*, increases by:
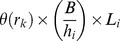
where *θ* is the probability of ingested L3 larvae establishing and becoming immature larvae in the host, and is a monotonic non-increasing function of *r*, representing the detrimental effect of resistance on parasite establishment.

Roberts and Grenfell [7] modelled a host resistance mechanism in which the level of resistance of host *k*, here denoted *r_k_*, was a function only of the number of L3 ingested. In reality, helminth populations are regulated by multiple density-dependent mechanisms [65], [66]. The acquisition of resistance is partially dependent on cumulative larval intake [67], [68]. However, adult burden also plays an important role in density-dependent regulation [50]. If resistance acquisition in the model were solely dependent on ingested L3, then the true impact of host grazing behaviours that delay the ingestion of L3 could not be explored. Consequently the model presented here has scope for mounted resistance to be dependent on the history of both L3 ingested and the number of established parasites.

When infective larvae are ingested, the resistance *r_k_* of host *k* increases by:
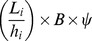
where *ψ* is a resistance gain coefficient. *r_k_,* also increases as a function of the current parasite burden, at rate (*a_k_*+*A_k_*)*η*, where *η* is a second resistance gain coefficient. Death of immature parasites in the host occurs at a rate ζa*_k_*. Immature parasites develop into mature, egg producing adult parasites at a rate χa_k_. Death of adults in host *k* occurs at rate *τ*(*r_k_*)*A_k_*, where *τ*(*r_k_*) >0 is a monotonic non-decreasing function which models the influence of acquired immunity on parasite mortality in the host. The loss of resistance in host *k* occurs at rate *σr_k_*.

*e_k_* represents the number of eggs in host *k*. Egg production from the dioecious parasites within host *k* occurs at a rate of:
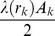
where *λ*(*r_k_*), the rate of egg production of adult parasites, is a monotonic non increasing function of *r_k_*.

The rate of defecation for an individual in its current patch is 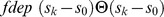 where the Heaviside function 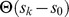 is unity if the stomach contents, *s_k,_* are greater than the faecal deposit size, *s_0_*, and is otherwise zero. When a defecation event occurs, e*_k_* decreases by 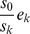 and the number of pre-infective larvae in patch *i, l_i_*, increases by the same quantity. The non-infective *l_i_* larvae develop into infective *L_i_* larvae on pasture at a rate of ε*l_i_*. The decay rate for faecal contamination at patch *i* is *φf_i_*, and the death rates of L and L3 larvae are *ωl_i_* and *ρL_i_* respectively. The stochastic model is simulated on the state-space variables (table 1) using the events and associated rates described above (see table 2) following the Gillespie algorithm [69]. Model parameters are listed in table 3.

**Table 2:** Summary of patch events, and animal events.

Patch Event	Rate r*_ei_*	Change in state variables
Growth of sward at patch *i*	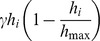	*h_i_ → h_i_* +1
Development of larvae at patch *i*:	ε*l_i_*	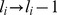 , 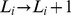
Death of pre-infective larvae at patch *i*:	*ωl_i_*	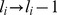
Death of infective L3 at patch *i*:	*ρL_i_*	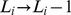
Decay of faeces at patch *i*:	*φf_i_*	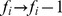
**Animal Event**	**Rate r** ***_ek_***	**Change in state variables**
Bite at current patch *i,* potential ingestion of infective and non-infective larvae, potential establishment of infective larvae and gain in immunity	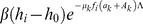	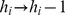 , 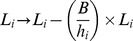 , * 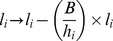 , 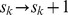 , 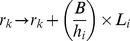 , 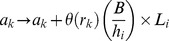 *
Death of immature adults in host *k*	ζa*_k_*	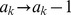
Maturity of adults in host *k*	χa_k_	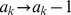 , 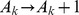
Death of adults in host *k*	*τ*(*r_k_*)*A_k_*	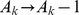
Gain of immunity in host *k* due to parasite burden	(*a_k_*+*A_k_*)*η*	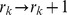
Loss of immunity in host *k*	*σr_k_*	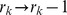
Egg production in host *k*	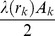	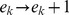
Defecation by host *k*	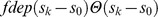	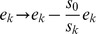 , 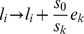 , 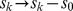 , 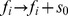
Movement of animal *k*	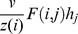	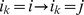

**Table 3:** Summary of parameters for patches, and animals.

Patch Parameter	
Intrinsic growth rate of sward	*γ*
Development rate of L to L3 larvae	ε
Death rate of pre-infective larvae (L)	*ω*
Death rate of L3 larvae	*ρ*
Decay of faeces	*φ*
**Animal Parameter**	
Bite rate	*β*
Faecal avoidance coefficient	*μ*
Death of immature larvae in host	ζ
Maturity of larvae in host	χ
Rate of resistance loss	*σ*
Resistance gain coefficient 1	*ψ*
Resistance gain coefficient 2	*η*
Death rate of adult larvae in host	*τ*
Rate of egg production of adult parasite	*λ(r_k_)*
Anorexia coefficient	*Λ*
Intrinsic movement rate	*V*
Probability of ingested L3 larvae establishing as adults	*θ (r_k_)*

All parameters expressed in units of minute^−1^, with the exception of *μ, ψ, η, Λ, r* and *θ*, which are dimension free.

### Parameterisation

Where parameter values are not stated for specific simulations, parameter values detailed in this section are used. The model was parameterised to simulate five hosts over one grazing season, in a set-stocked temperate grassland system, as described by Smith et al. [24]. All simulations were run for 365 days and replicate the spatial scale of such agricultural systems, using a field represented by a lattice consisting of 78×78 patches with each patch representing 0.5 m^2^. This patch area corresponds with the area of one faecal pat and the refusal zone around it [51]. Hosts move around the lattice with a search rate representative of a cattle step rate of approximately three steps per second [70] (*v* = 0.015), and a bite rate representing approximately 20,000 bites per day [51] (*β* = 0.1). When a bite event occurs, one unit of forage is removed. Each 0.5 m^2^ patch contains 50 bite areas of forage, as each cattle bite is approximately 0.01 m^2^ [51]. Each patch is initialised with a sward height that provides 200 units of forage, and has a maximum sward height providing 400 units of forage. Each patch has an ungrazeable portion of 50 units of forage, and grass grows over time at rate *γ* = 0.00004 [10]. These parameter values give rise to a set stocking scenario where intake approximately matches sward growth. Cattle deposit faeces approximately 10–15 times per day [51] (*f_dep_* = 1.0, *S_0_* = 2000.0), and the field is initialised with no faecal contamination (*f_i_*_ = _0 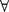 *i* = 1, …, *N*). Faeces decays at a rate where 10% of the faecal deposit remains 3 months post deposition [71] (*φ* = 0.00001776). Faecal avoidance for animal *k* varies from no avoidance (*μ_k_* = 0) to effectively complete avoidance (*μ_k_* = 10) [24], [52], where almost complete avoidance of fresh faeces (*μ_k_* = 5) results in a bite rate from freshly-contaminated patches <1% of the bite rate from non-contaminated patches [24], [52]. With these parameter values, the model reproduces the grazing behaviour that is empirically observed at multiple scales in livestock grazing from small scale choice experiments [40], [17], to large scale natural systems [54], [23].

The parasite’s lifecycle is representative of a typical gastrointestinal helminth of grazing herbivores in a temperate climate, with the extensive study of GIN lifecycles allowing the model to be meaningfully parameterised [4], [33]–[36]. Death rate of pre-infective stages (*ω* = 0.0001) results in approximately 1% of larvae remaining after 1 month [4], [35]. Approximately 50% of surviving pre-infective larvae develop to the infective L3 stage after 2 weeks on pasture [4], [33]–[35] (ε = 0.00005). The death rate of infective L3 results in approximately 10% remaining after 3 months [35], [36] (*ρ* = 0.000015).

Following ingestion of the infective stages approximately 40% of L3 larvae establish within a naïve host [35] (*p* = 0.4). The proportion that establish is monotonically non-increasing with increased levels of acquired resistance. Increase in resistance is dependent upon ingestion of L3 (*ψ = *0.25) and the size of the host’s parasite population (*η* = 0.025). In the absence of parasitism, immunity wanes over time (*σ* = 1.9×10^−8^) [7]. Ingested larvae develop into fecund adult parasites in approximately 3 weeks [35] (χ = 0.00003). Fecund adult parasites produce eggs at a rate which is monotonically decreasing as host resistance increases [35] (*λ* = 2). The life expectancy of the adult parasites in the host is approximately 5 weeks [35] (*τ* = 0.00002). With these parameter values, the model successfully reproduces the parasite dynamics empirically observed in livestock grazing systems [7], [47]–[50].

The starting condition of each simulation was representative of naïve hosts being released onto contaminated pasture. Each simulation was initialised with five uninfected hosts (*a_k_*_ = _0, *A_k_*_ = _0, 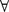 *k* = 1…D) on a pasture with 24000 infective L3, distributed over 20 randomly selected patches to reflect the aggregated distribution of larvae on pasture [9]. Each scenario was repeated over ten realisations to account for the stochastic nature of the model. The number of runs and herd size were limited by the extensive computational time required for this event based model. However, the size of the standard deviations in the results show that this number of runs was sufficient, and the findings of the grazing model have previously been shown to be robust in simulations based on herd sizes smaller than those used here [23], [24].

### Model Runs Performed

#### Aggregation of risk on pasture

Pre-infective larvae are released with host faeces, so aggregation of faeces results in uneven distributions of parasitic larvae on pasture. Cattle normally deposit faeces approximately 10–15 times per day [51] (*f_dep_* = 1.0, *s_0_* = 2000.0). To investigate the impact of aggregation of faeces and infective on-pasture larvae on parasite burden, simulations were run with varying sizes of faecal deposit (*s_0_*). Simulations were run with the number of faecal deposits ranging from 200 to 5 per day (*s*_0_ = 100, …, 4000, in increments of 100). Faecal avoidance was set at *μ_k_* = 3.

#### Influence of faecal avoidance across parasites with different development rates

There is substantial inter-species variation in observed larvae development rates [34], [35]. For GINs of herbivores in temperate climates, development times vary from less than one week to over five months [4], [33]–[35]. The influence of faecal avoidance behaviour on parasite transmission will vary with larval development time due to changes in the number and timing of larvae ingested. To investigate how faecal avoidance influences transmission of parasites with different on-pasture development times, simulations were run with varying development rates, *ε* = 0.00003 (development time of 3 weeks), *ε* = 0.00005 (development time of 2 weeks) and *ε = *0.0001 (development time of 1 week), over differing faecal avoidance levels ranging from no avoidance (*μ_k_* = 0), to effectively complete avoidance (*μ_k_* = 10).

#### Influence of faecal avoidance across hosts with different rates of resistance acquisition

A host’s ability to mount an effective immune response varies with factors such as the parasite species, host age, genotype, nutritional and hormonal status [27]. Simulations were run to determine how faecal avoidance influences parasite burden for parasite-host combinations where hosts have varying abilities to mount an immune response interpreted here in terms of rates of acquisition of immune resistance. Four sets of simulations were run for cohorts of hosts with: very low resistance (*ψ* = 0.01, *η = *0.0075), low resistance (*ψ = *0.125, *η = *0.0125), medium resistance (*ψ = *0.25, *η = *0.025), and high resistance (*ψ = *0.5, *η = *0.05). For each resistance level, simulations were run over differing faecal avoidance levels ranging from no avoidance (*μ_k_* = 0), to complete avoidance (*μ_k_* = 10).

#### Parasite-host behaviour interactions (parasite induced anorexia)

To elucidate the fundamental dynamics of the system, initial runs were performed with no explicit interaction between the host’s parasitised state and its behavioural response (*Λ* = 0). However hosts can have phenotypic plasticity, with parasitised animals exhibiting heightened faecal avoidance compared to non-parasitised animals [8], [16], [17], [24], [40], [52]. A set of simulations were run for a cohort of hosts with parasite-induced anorexia, where faecal avoidance 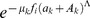 ranged from low to high depending on parasite burden (with *μ_k_* = 3, *Λ* = 0.0006, such that min *μ_k_* = 3, max *μ_k_* = 8, and mean *μ_k_* = 4). When parasite burden was highest, these hosts exhibited realistic levels of reduction in intake of approximately 40% compared to control hosts (*Λ* = 0) with low faecal avoidance (*μ_k_* = 3) [41]–[43]. For comparison, three further sets of simulations were run for cohorts of hosts with faecal avoidance level constant across the grazing season, at levels equivalent to the minimum, mean and maximum faecal avoidance levels exhibited by anorexic hosts; low faecal avoidance (*μ_k_* = 3, *Λ* = 0), high faecal avoidance (*μ_k_* = 8, *Λ* = 0), and average faecal avoidance (*μ_k_* = 4, *Λ* = 0).

### Quantities Observed in the Simulations

 peak parasite intensity, day of peak parasite intensity, and mean daily intake. Peak parasite burden was used as a measure of infection, as host morbidity and mortality are directly proportional to parasite intensity [72]. However, a host can be affected by both parasite intensity and duration of infection. To determine the usefulness of this measure as a reliable indicator of disease levels, both the peak parasite intensity and the cumulative exposure over the grazing season, measured by integrating the infection curve, were calculated for the scenarios detailed above. Over the range of simulations, both measures provided qualitatively similar results. Peak parasite intensity is used as a measure of infection here as it is a more intuitive measure than the area under the curve, and can be compared to empirical data. If cumulative burden was chosen instead as a measure of parasitism, the trends shown in the results, and the conclusions, would remain the same.
